# Cure for Ivy Poisoning

**Published:** 1883-01

**Authors:** 


Cure for Ivy Poisoning.—Bathe the parts affected with sweet spirits of nitre. If the blisters be broken, so as to allow the nitre to penetrate the cuticle, more than a single application is rarely necessary ; and even where it is only applied to the surface of the skin three or four times a day, there is rarely a trace of the poison left next morning.

